# Clinical Research of Lupus Retinopathy: Quantitative Analysis of Retinal Vessels by Optical Coherence Tomography Angiography in Patients with Systemic Lupus Erythematosus

**DOI:** 10.3390/diagnostics13203222

**Published:** 2023-10-16

**Authors:** Ximin Wang, Huan Xie, Yao Yi, Jinhan Zhou, Huimin Yang, Jin Li

**Affiliations:** Department of Ophthalmology, School of Medicine, Affiliated Renji Hospital, Shanghai Jiao Tong University, Shanghai 200120, China; wangximin3366@163.com (X.W.); xiehuan1997@163.com (H.X.); yiyao4629@163.com (Y.Y.); z1051416102@163.com (J.Z.)

**Keywords:** retinal vessels, lupus retinopathy (LR), systemic lupus erythematosus (SLE), optical coherence tomography angiography (OCTA)

## Abstract

Background: Lupus retinopathy, an ocular manifestation of systemic lupus erythematosus (SLE), is the major pathology attributed to retinal vasculopathy. Our study is to analyze the changes in retinal vessels in patients with SLE by optical coherence tomography angiography. Methods: A total of 61 SLE patients without obvious retinal manifestation and 71 healthy people were included. The SLE patients were further divided into a lupus nephritis (LN) group and a non-LN group. The changes in central macular thickness (CMT) and the retinal vessel densities were compared between the two groups, and the correlation between retinal vascular changes and disease activity was analyzed. Results: Compared with healthy control, the CMT and the retinal vascular densities in both superficial and deep retina were decreased significantly in SLE patients. There was no significant difference in retinal vascular densities between LN groups and non-LN groups. Conclusion: The CMT and retinal vessel densities were decreased in SLE patients without clinical manifestations, which might serve as a sensitive biomarker for early changes of lupus retinopathy in SLE patients.

## 1. Introduction

Systemic lupus erythematosus (SLE) is a chronic immune disease involving multiple organs and seriously affects the quality of life. The SLE prevalence rate is estimated to be 0.02–0.15%, and 90% of the patients are women of childbearing age [1]. About 1/3 of SLE patients have ocular manifestations, including abnormalities of the adnexa, cornea, iris, retina, choroid and optic nerve [2]. Keratoconjunctivitis sicca is the most common ocular manifestation while retinal and choroidal involvement is most closely related to visual acuity. Lupus retinopathy (LR) is a potentially blinding ocular manifestation of SLE, with the prevalence ranging from 3% to 29% [2]. The main pathological changes of LR are attributed to retinal vasculopathy, more specifically, immune complex-mediated microangiopathy. Immune complex deposition and vascular injury cause complement activation, thus aggravating vascular injury [3]. In addition, lupus anticoagulants and antiphospholipid antibodies may cause micro-embolism of retinal blood vessels, resulting in microcirculation disorders [4]. Initially, LR presents with hemorrhage spots, cotton wool spots, arteriolar stenosis, and dilatation of capillaries and veins, which are the typical manifestations of microcirculation disturbance. Central retinal vein occlusion and central retinal artery occlusion are rare in LR but with poor prognosis and often resulting in irreversible visual impairment. Very few patients develop Purtscher-like retinopathy characterized by extensive microembolization in small vessels and micro-infarction. In a nutshell, LR is a kind of retinal vasculopathy.

The involvement of retinal vessels in patients with SLE may precede the symptoms of other organs and may be related to the activity phase of the disease [5]. Eighty-eight percent of patients with LR have active systemic disease, indicating that the occurrence of ocular fundus abnormalities, especially retinopathy, is a possible indication of disease activity [6]. LR may have potential pathological correlations with other organs affected by SLE. Seth et al. found that SLE patients with LR had higher rates of renal and neuropsychiatric impairments than those without retinal involvement [7].

Optical coherence tomography angiography (OCTA) is a novel ophthalmic imaging technology facilitating the evaluation of the retina under different conditions, such as diabetes, chronic kidney disease and retinal vein occlusion, and provides a non-invasive alternative for viewing details of microvessels [8,9,10]. It detects blood flow in capillaries by measuring changes in OCT signals in successive cross-sectional images taken at the same location to, reconstruct a three-dimensional blood vessel structure. As a novel, non-invasive and non-contact technology, OCTA has higher safety, higher speed, and wider clinical applications than those of fluorescein fundus angiography and indocyanine green fundus angiography. It can reveal the subclinical changes in the retina, which fails to be detected under a slit lamp. Thus, OCTA can be employed to monitor the changes in retinal microcirculation in patients with SLE. Several studies have reported that retinal microcirculation was decreased in SLE patients using OCTA [1,11,12,13,14]. However, there are several shortcomings, including the small sample size, incomprehensive assessment of the disease activity, etc.

LR is a retinal vascular disease affecting the visual quality of SLE patients to varying degrees. Although the onset of LR is correlated with the disease activity of SLE to a certain extent, the relationship between the change of retinal vascular density and the disease activity in the subclinical stage of LR is still unclear. It remains to study whether the various organ damage subtypes of SLE have similar retinal manifestations and whether the early changes of the retinal vessels can indicate the involvement of other organs. The aim of this study was to explore the effect of SLE on retinal vessel density, the correlation between disease activity and the change in retinal vessel density, and the relationship between other organs and the retina by using OCTA.

## 2. Materials and Methods

### 2.1. Research Subjects

In this study, the right eyes of 61 patients with SLE without clinically confirmed retinopathy and the right eyes of 71 age and sex-matched healthy controls (HC) were enrolled from 2018 to 2021 in the Shanghai Jiao Tong University School of Medicine Affiliated Renji Hospital, and all patients were diagnosed as SLE according to the American College of Rheumatology (ACR) criteria [15]. This study adhered to the Declaration of Helsinki and was approved by the Medical Ethics Committee of Renji Hospital, Shanghai Jiao Tong University School of Medicine (reference number: 2018-155). Informed consent was obtained from each subject involved in the study.

Inclusion criteria for SLE patients: (1) age ≥16 and ≤65 years, (2) diagnosed as SLE, (3) capable of cooperating with the examinations.

Exclusion criteria for SLE patients: (1) a history of glaucoma or ocular hypertension; (2) pathologic myopia (SE < −6 diopters); (3) ocular trauma or ophthalmic surgery involving the fundus; (4) fundus lesion visible (the common characteristics such as cotton wool spots, microaneurysms, hard exudate, neovascularization, hemorrhage, etc.) under ophthalmoscope; (5) diabetes, hypertension or other systemic diseases that cause retinopathy; and (6) unable to get high quality of OCTA images (signal quality index ≥ 5) due to cloudy refracting media or other reasons.

Inclusion criteria for HC: (1) age ≥16 and ≤65 years; (2) capable of cooperating with the examinations. 

Exclusion criteria for HC: (1) a history of SLE or the typical symptoms of SLE; (2) ocular disease; (3) pathologic myopia; (4) ocular trauma or ophthalmic surgery that involved fundus; (5) fundus lesion visible under ophthalmoscope; (6) diabetes, hypertension or other systemic diseases that cause retinopathy; and (7) unable to get high quality of OCTA images (signal quality index ≥5) due to cloudy refracting media or other reasons.

Patients were divided into the lupus nephritis (LN) group and the non-LN group according to renal injury. The LN group met the diagnostic criteria of the 2012 ACR guidelines for LN [16].

### 2.2. Ophthalmological Evaluation

**Figure 1:**
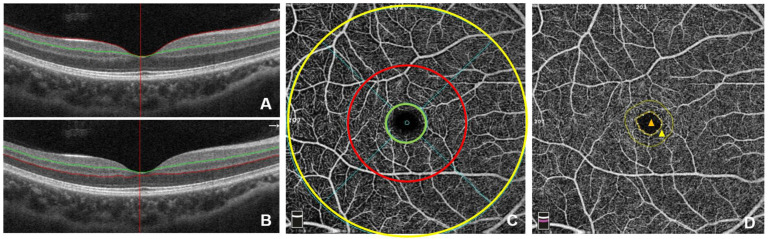
The schematic examination of the macula by optical coherence tomography angiography (OCTA). The macula was segmented into superficial retina (**A**), from ILM to IPL, and deep retina (**B**), from IPL to OPL. The enface of OCTA (**C**) showed the macular region was outlined according to the study of ETDRS, in which the green ring (1 mm diameter) is designated as foveal region, the area between the green ring and the red ring (3 mm diameter) is designated as parafoveal zone, and the area between the red ring and the yellow ring (6 mm diameter) is designated as peripheral fovea zone. (**D**) The area in the dotted yellow line is foveal avascular zone (FAZ, orange triangle), and the circular area between the solid yellow line and the dotted yellow line represents foveal density 300 μm (FD-300), i.e., the vessel density of the full-thickness retina in a width of 300 μm around the FAZ (yellow triangle).

All subjects received an ophthalmological examination, including slit-lamp examination, ophthalmoscopy and OCTA, and patients with SLE were examined additionally with a non-contact tonometer and best-corrected visual acuity (BCVA). All OCTA examinations were conducted by the same examiner applying the AngioVue OCTA system (Avanti RTVue-XR; Optovue, Fremont, CA, USA). The macula of the retina was examined using OCTA focusing on the fovea by Retina 6.0 mode and segmented into superficial retinal vessels (Figure 1A), from the internal limiting membrane (ILM) to inner plexiform layer (IPL), and deep retinal vessels (Figure 1B), from IPL to outer plexiform layer (OPL). The retinal microvessels of the superficial retina and deep retina were obtained on the enface of OCTA (6 mm × 6 mm), and were divided into several regions (Figure 1C,D). The quality of the image was strictly controlled, and only high-quality scans (signal quality index ≥ 5, range from 0−10) were used for analysis. Manual correction was necessary when the different retinal layers failed to be identified. 

The parameters used to evaluate the retina included the central macular thickness (CMT), the size of the foveal avascular zone (FAZ), foveal density 300 μm (FD-300), as well as the vascular densities in the whole, foveal region, parafoveal zone and perifoveal zone in both superficial and deep retina.

### 2.3. Rheumatologic Assessment

For patients with SLE, disease activity was assessed by the Systemic Lupus Erythematosus Disease Activity Index 2000 (SLEDAI-2K) [17] and several items in SLEDAI-2K, including complement (C3 and C4), white blood count (WBC), platelet count (PLT) and anti-double-stranded DNA antibody (anti-dsDNA).

### 2.4. Statistical Analysis

Kolmogorov–Smirnov One-sample Test was used to test the normality of data sets. Normally distributed variables were represented as mean ± standard deviation (SD). Non-normally distributed variables were represented as median. Categorical variables were described with absolute frequencies and percentages. The chi-square test was used to compare the gender composition between the two groups. OCTA data in different two groups were compared using the *t*-test or Mann–Whitney U test as required. Pearson correlation test (for normally distributed variables) or Spearman’s rank (for non-normally distributed variables) were used to determine the significance of the correlation between the OCTA parameters and laboratory data. *p* values < 0.05 were considered statistically significant. All statistics were analyzed by the statistical package SPSS 21.0 and GraphPad Prism (version 8; GraphPad software).

## 3. Results

### 3.1. Comparison of Retinal Vascular Parameters between SLE Patients and HC

In this study, we enrolled 61 patients with SLE, including 7 males and 54 females, with an average age of 36.79 years old. A total of 71 healthy subjects were enrolled, including 11 males and 60 females, with an average age of 36.63 years old. There was no significant difference in age (*p* = 0.935) and gender (*p* = 0.502) between the two groups. Among the 61 SLE patients, the duration of SLE ranged from 1 to 240 months, with a median of 48 months. Only 5 of the 61 SLE patients (8.20%) were diagnosed with neuropsychiatric systemic lupus erythematosus, and nearly half of the patients (47.54%) were diagnosed with LN.

**Figure 2:**
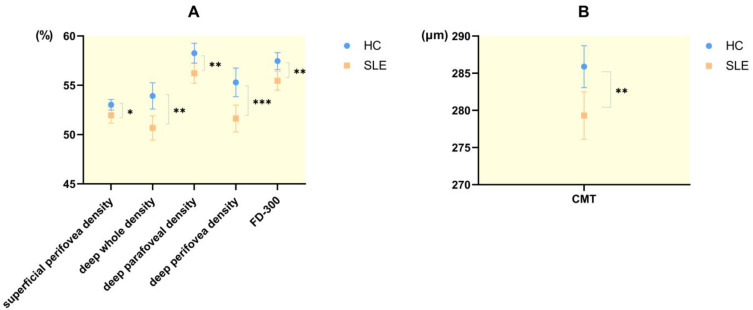
The changes in retinal vessel densities and central macular thickness (CMT) between patients with SLE and HC. (**A**) The FD-300, superficial perifovea density as well as vessel densities of the whole, parafoveal zone and perifoveal zone in deep retina in SLE patients were decreased significantly compared with that in HC. (**B**) The CMT in SLE patients was decreased significantly compared with that in HC. * *p* < 0.05. ** *p* < 0.01. *** *p* < 0.001.

The CMT, FD-300, the superficial perifovea density as well as the vessel densities in the whole, parafoveal zone and perifoveal zone in the deep retina in SLE patients were lower than those of HC, and the differences were statistically significant (Figure 2). There was no significant difference in FAZ, deep foveal vessel density and the vascular densities in the whole, foveal region and parafoveal zone in the superficial retina between the two groups (Table 1).

### 3.2. Correlation between Retinal Vessel Densities and Disease Activity in SLE Patients

The laboratory parameters of SLE patients and normal range are shown in Table 2. Nearly half of the patients were anti-dsDNA positive. The mean concentration of C3 in patients with SLE was below the normal range, while the mean concentration of C4, WBC and PLT levels were in the normal range.

**Figure 3:**
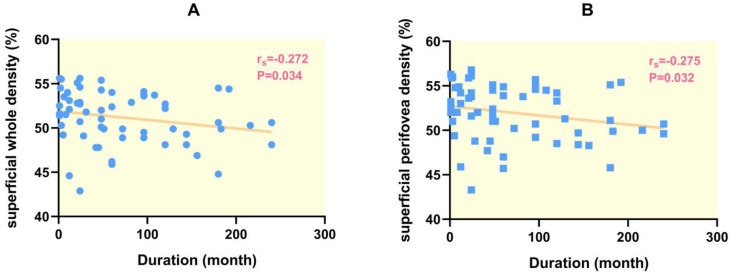
Correlation between duration of SLE and retinal vessel density. Duration of SLE was negatively correlated with superficial whole enface vessel density (**A**) and superficial perifoveal vessel density (**B**). r_s_ Spearman’s rank.

The disease duration was negatively correlated with retinal vessel densities in the whole and perifoveal zone in the superficial retina (Figure 3), but was not correlated with FAZ size, FD-300, CMT, superficial foveal vessel density, superficial parafoveal density as well as retinal vessel densities in the whole, foveal zone, parafoveal zone and perifoveal zone in the deep retina.

Retinal vessel density, FAZ size and CMT in SLE patients were not correlated with SLEDAI, C3, C4, WBC, PLT and anti-dsDNA (*p* > 0.05, Table S1: the *p* value of correlation analysis between OCTA data and rheumatologic data related to disease activity). 

### 3.3. Correlation between Retinal Vessel Density and LN in SLE Patients

The patient information and the corresponding retinal vessel densities and CMT in both LN group and non-LN groups are shown in Table 3. The age and gender composition and disease duration of these two groups were not significantly different from each other. There was no significant difference in retinal vessel densities nor CMT between the LN group and non-LN group. OCTA data of the LN group and non-LN group were not correlated with SLEDAI, C3, C4, WBC or PLT. 

## 4. Discussion

In the course of LR, the symptoms of patients change from mild to severe, and the lesions also develop from localized to extensive, showing a trend of slow aggravation. Thus, there might be subclinical pathological changes in LR, and the cumulative effect of these changes consequently leads to clinical manifestations. Kim et al. [18] found similar changes as seen in LR in patients who are diabetic, and the changes in microcirculation in diabetic patients may precede clinically distinguishable retinopathy. We hypothesized that such subclinical changes in retinal microcirculation may also be present in SLE patients. In a study of OCT images of 26 patients (52 eyes) with SLE, retinal changes in SLE patients on OCT mainly included degenerative thinning, foveal flattening, epiretinal membrane and incomplete posterior vitreous detachment [19]. In our study, SLE patients without obvious clinical manifestations of LR had lower retinal vessel density and thinner CMT than those in the healthy subjects, suggesting that changes in retinal microvessels may precede the clinical manifestation of LR.

The application of OCTA brings the exploration of retinal microvascular diseases to a higher level. In recent years, more and more scholars have used OCTA to conduct in-depth research on LR based on the microvascular level. A prospective, cross-sectional study comparing SLE patients without macular injury and healthy controls found significant differences in microvascular structure by OCTA examination [20]. Shi et al. [11] found that the microvessel density in some regions of superficial and deep layers in SLE patients was significantly reduced. Other studies have found that visual acuity, retinal thickness, and retinal vascular density in SLE patients are significantly lower than those in HC, and there is a certain association between these factors [1]. The study excluded SLE patients with retinopathy or choroidal diseases, but did not specify whether patients with microvascular manifestations such as cotton-wool spots. Francesco et al. [21] found that even without ocular symptoms, SLE patients had lower superficial vascular density, especially in the parafoveal region, and larger FAZ compared with healthy people, but there was no significant difference in deep retinal vessels. However, in our study, the retinal vessel densities in both superficial and deep retina were significantly decreased in SLE patients than in healthy subjects, with no significant difference for FAZ between the two groups. This may be due to differences in sample size, disease duration, race, instrumentation, and measurement methods. The conclusions of other research support our results, suggesting that the FAZ area of SLE patients was not different from that of healthy people, while the retinal vessel densities in superficial and deep retina as well as FD-300 in SLE patients were decreased [12].

The macular region contains four retinal vascular networks, including radial peripapillary capillary plexus (RPCP), superficial vascular plexus (SVP), intermediate capillary plexus (ICP) and deep capillary plexus (DCP) [22]. At present, the superficial retinal vessels include RPCP and SVP, and the deep retinal vessels include ICP and DCP, as defined in OCTA. Superficial vessels are extremely dense in the peri-optic disc area, and the density decreases along the macular papillary axis with distance from the disc. There was no distinct difference in the density of vessels near the fovea and around the fovea in DCP. The capillary density of ICP decreases gradually from the fovea to the periphery, plummeting at 4 mm away from the fovea and then disappearing at 8mm of eccentricity [23]. The boundary of ICP is usually the area where diabetic retinopathy develops, and we speculate that SLE-related changes in retinal microcirculation may also start there. We found no difference in FAZ size between SLE patients and HC, and our results showed that the course of SLE was inversely related to superficial vessel density but not to foveal or parafoveal vessel density, consistent with the hypothesis that the lesion starts in the periphery. The superficial retinal capillary density of SLE patients treated with hydroxychloroquine is lower than that of non-users, and the superficial capillary density of SLE patients taking hydroxychloroquine for more than 5 years is lower than that of those taking hydroxychloroquine for less than 5 years [24]. Given the findings that the disease duration is related to the superficial retina and that both the superficial and deep retinal vessels are affected in the course of SLE, we hypothesized that the superficial capillaries may be the primary target vessels attacked by drugs (hydroxychloroquine, chloroquine, glucocorticoid, etc.) or disease progression in SLE.

Retinal vessel is directly visible in vivo, and often reflect the degree of systemic vascular injury [11]. The most common pathological type of renal injury in SLE is glomerulonephritis, while renal vasculitis is a rare subtype [25,26]. In a multicenter study of 285 renal biopsy samples from patients with LN, only 27% of the cases had renal vascular lesions, and 2.8% had vasculitis [27]. Conigliaro et al. [14] demonstrated that compared with patients without nephritis, the density and thickness of parafoveal vessels were reduced in patients with nephritis. Mehmed et al. [20], with a sample of 35 SLE patients, could not find any differences in CMT, FAZ and vessel density between the two groups of patients with and without LN. A cross-sectional study of Conigliaro reported the correlation between retinal vascular data and the LN-vascular lesions in kidney biopsy, and LR was present in 56.5% of LN patients [28]. The results of our study showed no significant difference in OCTA data between LN and non-LN patients without retinopathy, confirming that renal vascular injury may not be directly related to retinal vascular density at the subclinical stage of LR.

Retinopathy in SLE patients mostly occurs in the active stage of the disease [4]. SLEDAI, C3, C4, WBC, anti-dsDNA and PLT can reflect the disease activity of SLE to some extent. SLEDAI score, as a recognized evaluation criterion for SLE disease activity, has been included in several studies. SLE patients with retinopathy had significantly higher SLEDAI scores than those without retinopathy, and most patients with retinal microangiopathy were in the systemic active phase [4,7]. Conigliaro et al. found an inverse association between SLEDAI score and retinal vessel density in SLE patients [14]. Other studies had the same result as ours; that is, the SLEDAI score was not correlated with retinal vessel density [11,12]. Due to its complexity and subjectivity, the SLEDAI score has low sensitivity to clinical changes in the evaluation of SLE disease activity and has certain limitations, which cannot accurately reflect the condition of patients [29].

A previous study has shown that anti-dsDNA antibody plays an important role in the development of SLE and is involved in the classification and diagnosis of SLE [30]. Anti-dsDNA antibodies can not only distinguish between inactive and active phases but also predict disease progression and even monitor disease response to treatment [31]. In our study, there was no significant difference in retinal vascular parameters between anti-dsDNA positive and anti-dsDNA negative patients.

Abnormal WBC count may be related to SLE disease activity, drug toxicity and infection. Leukocytes include granulocytes, monocytes and lymphocytes, among which neutrophils are key effector cells in the pathogenesis of SLE, and low-density granulocytes (LDG), as a subgroup of neutrophils, are elevated in SLE [32]. LDGs spontaneously form proinflammatory neutrophil extracellular traps (NETs), which can promote thrombosis, inflammation and fibrosis, playing an important role in the progression of SLE [33]. LDGs are increasingly reserved in microvessels, which has important pathogenic implications in the context of microvascular diseases. We found no correlation between WBC and retinal vessels in the SLE group, LN group and non-LN group. It is necessary to explore the correlation between LDGs and retinal vascular changes in the future.

Measurement of complement is a recognized part of disease activity assessment in SLE and has been included in the new European League Against Rheumatism (EULAR)/ACR classification criteria. Although earlier studies have suggested that low complement levels may be associated with the activity of LN, it hardly happens in very early and mild diseases [34]. Complement activation cascade products, formed from activation of the complement cascade, can reflect complement activation more accurately than a single intact protein and are more closely related to disease activity in SLE patients [34]. We found no correlation between complement levels and retinal vascular structure in all SLE groups. Complement fragment level may be considered as an indicator of disease activity to analyze its correlation with retinal vessels in the future.

Although LR is likely to be associated with disease activity, retinal vascular density is not associated with disease activity in SLE patients without clinical retinal manifestations.

Limitations of our study include cross-sectional design, lack of analysis of drug treatment (prednisone, hydroxychloroquine, etc.), and not evaluating the SLICC (systemic lupus international collaborating clinics) damage index.

## 5. Conclusions

In our study, the decreased retinal vessel densities and CMT in OCTA indicated the early changes in the subclinical stage of LR, which might serve as a sensitive biomarker for LR. Retinal vascular densities were correlated with the duration of SLE but not correlated with disease activity in patients without retinal manifestations. Superficial retinal capillary may be the primary target effect in SLE. The occurrence of nephritis seems to be independent of changes in retinal vascular structure.

## Figures and Tables

**Table 1:** Comparison of retinal vessel densities and CMT between SLE patients and HC.

	SLE	HC	*p* Value
superficial whole density (%)	51.18 ± 2.97	52.05 ± 2.18	0.062
superficial foveal density (%)	18.86 ± 6.70	19.05 ± 6.27	0.866
superficial parafoveal density (%)	53.85 ± 3.31	54.79 ± 2.66	0.073
superficial perifovea density (%)	51.96 ± 3.10	53.03 ± 2.27	0.028 *
deep whole density (%)	50.68 ± 4.81	53.92 ± 5.62	0.001 *
deep foveal density (%)	34.07 ± 7.35	35.02 ± 7.10	0.455
deep parafoveal density (%)	56.22 ± 3.94	58.26 ± 4.25	0.005 *
deep perifovea density (%)	51.63 ± 5.33	55.29 ± 6.10	<0.001 *
FAZ (mm^2^)	0.318 ± 0.102	0.330 ± 0.105	0.503
FD-300 (%)	55.46 ± 3.74	57.46 ± 3.63	0.002 *
CMT (μm)	279.31 ± 12.44	285.89 ± 11.94	0.003 *

* Statistically significant. FAZ, foveal avascular zone; FD-300, foveal density 300 μm; CMT, central macular thickness.

**Table 2:** The clinical data of the SLE patients and the normal range.

Clinical Data	SLE	Normal Range
C3 (g/L)	0.748 ± 0.278	0.9–1.8
C4 (g/L)	0.134 ± 0.082	0.1–0.4
WBC (10^9^/L)	6.02 ± 2.76	3.97–10.15
PLT (10^9^/L)	212.33 ± 74.53	85–303
anti-dsDNA positive (n)	27 (44.3%)	
SLEDAI	6.93 ± 5.07	
disease duration (month)	48	

anti-dsDNA, anti-double-stranded DNA antibody; C3, complement 3; C4, complement 4; WBC, white blood count; PLT, platelet count; SLEDAI, systemic lupus erythematosus disease activity index.

**Table 3:** Basic data and retinal vascular data of lupus nephritis (LN) and non-LN group.

	NLN Group	LN Group	*p*
N	32	29	
age (year)	36.84 ± 12.01	36.72 ± 11.42	0.968
female	30 (93.8%)	24 (82.8%)	0.179
duration (month)	27.5	72	0.095
superficial whole density (%)	51.18 ± 3.41	51.19 ± 2.45	0.981
superficial foveal density (%)	18.43 ± 6.42	19.33 ± 7.07	0.602
superficial parafoveal density (%)	53.67 ± 3.88	54.05 ± 2.58	0.659
superficial perifovea density (%)	52.03 ± 3.49	51.88 ± 2.68	0.847
deep whole density (%)	51.12 ± 4.54	50.20 ± 5.12	0.459
deep foveal density (%)	33.85 ± 7.44	34.31 ± 7.37	0.809
deep parafoveal density (%)	56.63 ± 3.71	55.75 ± 4.20	0.387
deep perifovea density (%)	52.18 ± 5.00	51.02 ± 5.70	0.398
FAZ (mm^2^)	0.318 ± 0.100	0.317 ± 0.106	0.986
FD-300 (%)	54.96 ± 4.36	56.01 ± 2.90	0.279
MT (μm)	276.59 ± 10.55	282.31 ± 13.82	0.073

FAZ, foveal avascular zone; FD-300, foveal density 300 μm; CMT, central macular thickness.

## Data Availability

Raw data from this study are available from the corresponding author upon reasonable request.

## Supplementary Materials

The following supporting information can be downloaded at: https://www.mdpi.com/article/10.3390/diagnostics13203222/s1, Table S1: The *p* value of correlation analysis between OCTA data and rheumatologic data related to disease activity.
