# Supplementary material for: Clinical Research of Lupus Retinopathy: Quantitative Analysis of Retinal Vessels by Optical Coherence Tomography Angiography in Patients with Systemic Lupus Erythematosus
Source: Diagnostics (Basel). 2023 Oct 16;13(20):3222. doi: 10.3390/diagnostics13203222 (PMC10606127; doi:10.3390/diagnostics13203222)
Supplement: Supplementary file 1 [file diagnostics-13-03222-s001.zip › diagnostics-2593242-supplementary.pdf]

Table S1. The P value of correlation analysis between OCTA data and rheumatologic data related to disease activity

	SLEDAI	anti-dsDNA	C3	C4	WBC	PLT
superficial whole density	0.450	0.962	0.742	0.182	0.950	0.542
superficial foveal density	0.381	0.821	0.802	0.699	0.182	0.191
superficial parafoveal density	0.421	0.622	0.958	0.382	0.914	0.501
superficial perifovea density	0.458	0.995	0.748	0.202	0.833	0.561
deep whole density	0.370	0.303	0.421	0.604	0.343	0.417
deep foveal density	0.998	0.816	0.828	0.780	0.228	0.133
deep parafoveal density	0.262	0.093	0.204	0.403	0.314	0.749
deep perifovea density	0.462	0.318	0.505	0.689	0.325	0.286
CMT(μm)	0.829	0.351	0.441	0.064	0.793	0.749
FAZ	0.682	0.775	0.794	0.959	0.104	0.153
FD-300	0.727	0.642	0.692	0.907	0.776	0.929
